# Supplementary material for: Whole Exome Sequencing of Thymoma Patients Exhibiting Exceptional Responses to Pemetrexed Monotherapy
Source: Cancers (Basel). 2023 Aug 8;15(16):4018. doi: 10.3390/cancers15164018 (PMC10452868; doi:10.3390/cancers15164018)
Supplement: Supplementary file 1 [file cancers-15-04018-s001.zip › cancers-2555619-supplementary figures.pdf]

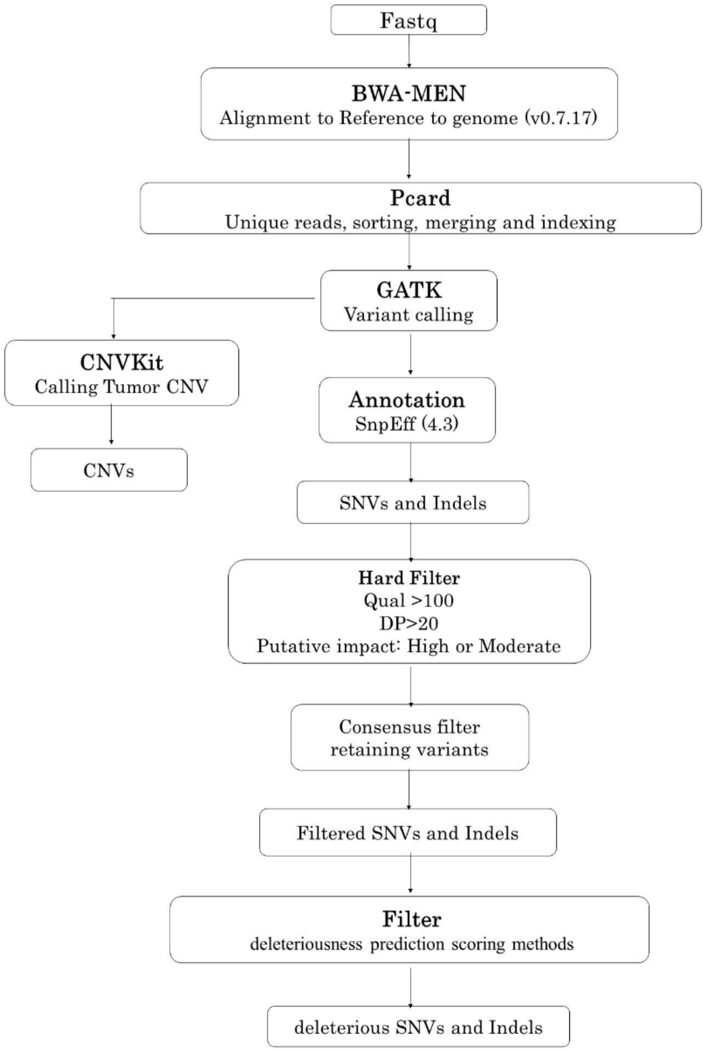

Figure S1: The bioinformatics analysis pipeline used in this study. Single-nucleotide variants (SNVs) and insertion and deletions (Indels) were analyzed using GATK. CNVkit was used for calling copy number variations (CNVs).

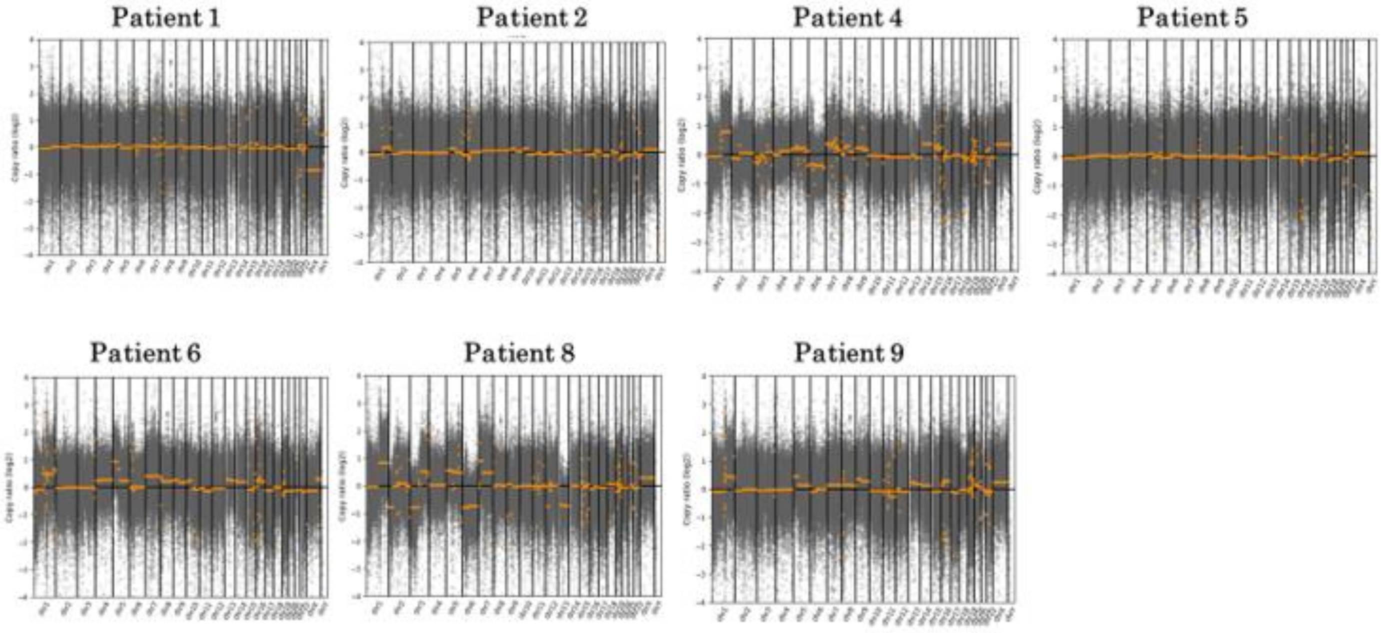

Figure S2: Scatter plots of copy number variations in thymomas.
